# Stress and the City: Measuring Effects of Chronic Stress and Air Pollution

**DOI:** 10.1289/ehp.118-a258b

**Published:** 2010-06

**Authors:** Ernie Hood

**Affiliations:** **Ernie Hood** is a science writer, editor, and podcast producer in Hillsborough, NC. He hosts a weekly science radio show, *Radio in Vivo*, and has written for *EHP* since 1999

Scientists have begun to consider an association between chronic psychosocial stress and increased susceptibility to adverse physiologic effects from exposures to air pollution. Although epidemiologic evidence tends to support that hypothesis, it has proven difficult to untangle the complex web of exposure effects, stressors, and mechanisms behind the potential differences in susceptibility. A new laboratory study documents different responses to air pollution in stressed and nonstressed rats, supporting the epidemiologic evidence that chronic stress may alter respiratory responses to air pollution **[*EHP*** **118:769–775; Clougherty et al.]**. The protocol described in the study may provide a template for future controlled experiments to explore associations between psychosocial and environmental factors.

The authors randomly divided 24 rats into four groups—stress plus exposure to uniform doses of concentrated ambient particulates (CAPs), nonstress plus exposure to CAPs, stress plus exposure only to filtered air (FA) from which particles were removed, and nonstress plus exposure to FA. Each animal in the two stress groups was individually put into the cage of a dominant male rat for 20 minutes at a time, twice per week; the older male behaved territorially and aggressively toward the younger test rodents, which were able to protect themselves from scratches and bites by retreating into a Plexiglas tube in the cage. On the day following each stress exposure, animals were exposed to either CAPs or FA for 5 hours. All exposures occurred at the same time each day to account for normal diurnal variation in the animals’ stress hormone and activity levels.

Respiratory responses were monitored continuously during the CAPs/FA exposure periods. Both CAPs-exposed groups had a significant response to the exposure, but the stress group exhibited greater breathing frequency, shorter inhalation and exhalation times, and lower expiratory flows and tidal volumes—that is, a rapid, shallow breathing pattern—compared with the nonstress group.

The researchers also observed changes in levels of several systemic inflammatory biomarkers associated with airway disease. Stress alone or CAPs exposure alone elevated some biomarkers, but only the group exposed to both stress and CAPs showed elevated levels of C-reactive protein and increased numbers of lymphocytes and monocytes, indicating the combination of exposures may have a different effect on inflammation than either exposure alone. This finding provides evidence that chronic stress may increase susceptibility to effects of air pollution on respiratory diseases.

As far as human exposures go, the authors note that social stressors (such as poverty and violence) and environmental exposures (such as traffic-related pollution) may be spatially correlated; “thus,” they write, “the most pollution-exposed communities may also be the most susceptible.” Although this study was small, it points wto important new ways to characterize the combined physiologic impact of those real-world phenomena.

